# Supplementary material for: Disrupted reward processing in Parkinson’s disease and its relationship with dopamine state and neuropsychiatric syndromes: a systematic review and meta-analysis
Source: J Neurol Neurosurg Psychiatry. 2021 Dec 20;93(5):555–62. doi: 10.1136/jnnp-2021-327762 (PMC9016258; doi:10.1136/jnnp-2021-327762)
Supplement: Supplementary data [file jnnp-2021-327762supp001.pdf]

Supplementary references for Table 1.

s1. den Brok, M. G. H. E. et al. Apathy in Parkinson's disease: A systematic review and meta-analysis. Mov. Disord. (2015). doi:10.1002/mds.26208
s2. Lemke, M. R. Anhedonia, Depression, and Motor Functioning in Parkinson's Disease During Treatment With Pramipexole. J. Neuropsychiatr. (2005). doi:10.1176/appi.neuropsych.17.2.214
s3. Husain, M. & Roiser, J. P. Neuroscience of apathy and anhedonia: A transdiagnostic approach. *Nature Reviews Neuroscience* (2018). doi:10.1038/s41583-018-0029-9
s4. Reijnders, J. S. A. M., Ehrt, U., Weber, W. E. J., Aarsland, D. & Leentjens, A. F. G. A systematic review of prevalence studies of depression in Parkinson's disease. *Movement Disorders* (2008). doi:10.1002/mds.21803
s5. Schrag, A., Ben-Shlomo, Y. & Quinn, N. How common are complications of Parkinson's disease? J. Neurol. 249, 419–423 (2002).
s6. Halahakoon, D. C. *et al.* Reward-Processing Behavior in Depressed Participants Relative to Healthy Volunteers: A Systematic Review and Meta-analysis. *JAMA Psychiatry* (2020). doi:10.1001/jamapsychiatry.2020.2139
s7. Broen, M. P. G., Narayen, N. E., Kuijf, M. L., Dissanayaka, N. N. W. & Leentjens, A. F. G. Prevalence of anxiety in Parkinson's disease: A systematic review and meta-analysis. Mov. Disord. 31, 1125–1133 (2016).
s8. Charpentier, C. J., Aylward, J., Roiser, J. P. & Robinson, O. J. Enhanced Risk Aversion, But Not Loss Aversion, in Unmedicated Pathological Anxiety. Biol. Psychiatry 81, 1014–1022 (2017).
s9. Drew, D. S. *et al.* Dopamine and reward hypersensitivity in Parkinson's disease with I mpulse control disorder. *Brain* **143**, 2502–2518 (2020).
s10.. Pezzella, F. R. et al. Prevalence and clinical features of hedonistic homeostatic dysregulation in Parkinson's disease. Mov. Disord. 20, 77–81 (2005).
s11. Luijten, M., Schellekens, A. F., Kühn, S., Machielse, M. W. J. & Sescousse, G. Disruption of Reward Processing in Addiction : An Image-Based Meta-analysis of Functional Magnetic Resonance Imaging Studies. JAMA Psychiatry 74, 387–398 (2017).
s12. Fénelon, G. & Alves, G. Epidemiology of psychosis in Parkinson's disease. *J. Neurol. Sci.* **289**, 12–17 (2010).
S13. Radua, J. *et al.* Ventral Striatal Activation During Reward Processing in Psychosis: A Neurofunctional Meta-Analysis. *JAMA Psychiatry* **72**, 1243–1251 (2015).

Supplement figure 1. Contour-Enhanced Funnel Plot of all studies Parkinson's versus healthy controls

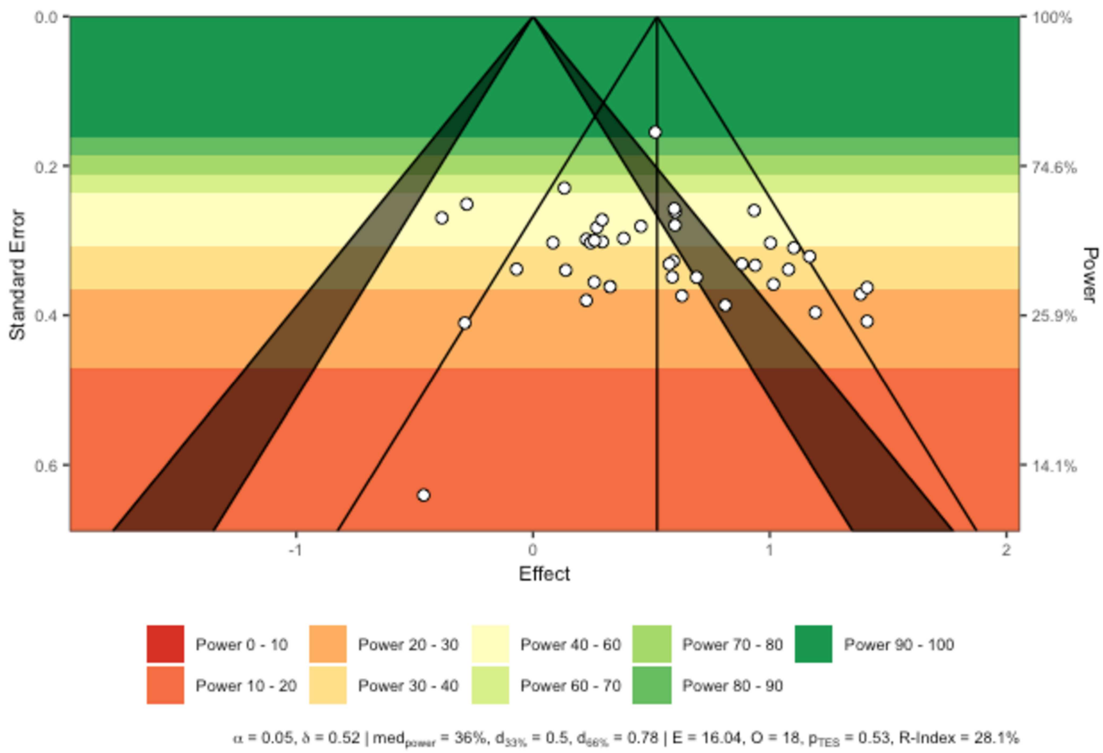

Supplement figure 2. Forest plot of reinforcement learning (RL) in Parkinson's patients with and without Impulse Control Disorder (ICD)

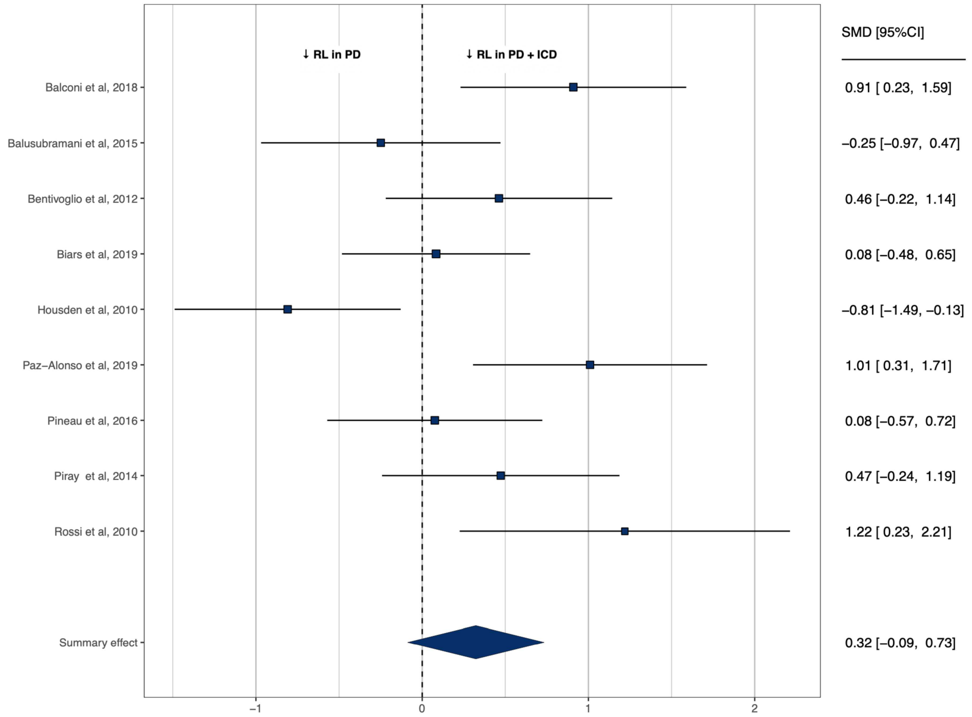

Formulae used to convert study measures into Cohen's ds and associated variances.

The following formulae were used to convert study measures into Cohen's ds and associated variances **between subjects:**

$$d = \frac{M_1 - M_2}{SD_{pooled}}$$

Equation 1. Cohen's d from Means and Standard Deviations of 2 samples. d is Cohen's d, M1 is the mean of one sample M2 is the mean of the other sample, SDpooled is the pooled standard deviation of the two samples (please see below.)

$$SD_{pooled} = \sqrt{(N_1 - 1)SD_1^2 + (N_2 - 1)SD_2^2}$$

Equation 2. Pooled standard deviation of 2 samples. SDpooled is the pooled standard deviation of the two samples, N1 is the size of one sample N2 is the size of the other sample, SD1 is the standard deviation of one sample, SD2 is the standard deviation of the other sample.

$$d = \frac{t}{\sqrt{\frac{1}{\frac{1}{N_1} + \frac{1}{N_2}}}}$$

Equation 3. Cohen's d from t-statistic. d is Cohen's d, N1 is the size of one sample N2 is the size of the other sample, t is the t-statistic

$$d = \frac{F}{\sqrt{\frac{1}{\frac{1}{N_1} + \frac{1}{N_2}}}}$$

Equation 4. Cohen's d from F-statistic. d is Cohen's d, N1 is the size of one sample N2 is the size of the other sample, F is the F-statistic.

$$Var_d = \frac{N_1 + N_2}{N_1 \times N_2} + \frac{d^2}{2(N_1 + N_2)}$$

Equation 5. Variance on Cohen's d for between subjects. Vard is the Variance on Cohen's d, N1 is the size of one sample N2 is the size of the other sample, d is Cohen's d.

The following formulae were used to convert study measures into Cohen's ds and associated variances **within subjects:**

$$d = \frac{M_d}{SD_d}$$

Equation 6. Cohen's d from Means and Standard Deviations from within subjects sample. Where M_d is the mean change and SD_d is the SD of the change scores
(equal to $SD_d = \sqrt{SD_1^2 + SD_2^2 - 2 \times r \times SD_1 SD_2}$).

$$d = \frac{t}{\sqrt{N}}$$

Equation 7. Cohen's d from t-statistic for within subjects results. d is Cohen's d, N is the sample size, t is the t-statistic

$$d = \sqrt{\frac{F}{N}}$$

Equation 8. Cohen's d from F-statistic. d is Cohen's d, N is the sample size, F is the F-statistic.

$$Var[d] = \frac{1}{n} + \frac{d^2}{2n}$$

Equation 9. Variance on Cohen's d for within subjects, n is the size of the sample.

Supplement table 1. Option Valuation study characteristics and participant demographics.

Option valuation in Parkinson's vs Healthy Controls				Age (years)			Disease duration (years)		UPDRS		Cognition			Depression		
Study	Task	Group	n	Mean	SD	% female	Mean	SD	Mean	SD	Cognitive measure	Mean	SD	Depression measure	Mean	SD
Bayard, 2016	Game of dice task	PD	78	67.49	8.16	35	7	.	24	(5-80)	MMSE	28.1	1.93	BDI	12.5	7.38
		HC	96	67.95	6.75	32	-	-	-	-		28.46	1.38		10.04	11.76
Brandt, 2015	Game of dice task	PD	15	64.78	8.09	46.66	.	.	14.43	10.14	MoCA	26.87	1.69	GDS-15	3.33	3.54
		HC	15	62.39	10.04	40	-	-	-	-		27.6	1.43		1.33	1.18
Chong, 2015	Effort based decision making task	PD	26	66.6	6.8	34.6	.	.	21.6	11.7	MoCA	28.2	1.3	DASS	2	2.23
		HC	26	66.2	6.4	42.3	-	-	-	-		28.2	1.7		1.5	1.84
Cools, 2003	Decision making gambling task	PD	12	64.6	1.5	58.33	6.5	1.4	30.9	6.8	MMSE	29.5	0.15	BDI	7.7	1.1
		HC	12	.	.	.	-	-	-	-		.	.		.	.
Kobayashi, 2019	Economic choice task	PD	15	63	6.58	40	6.93	6.2	39	14.6	MMSE	28.3	1.76	.	.	.
		HC	21	60	6.69	57.1	-	-	-	-		28.7	1.99		.	.
Le Bouc, 2016	Incentivised grip force choice task	PD	24	60.2	1.6	29.17	11.4	1.3	11.7	1.7	MMSE	27.3	1.3	MADRS	5.9	1
		HC	25	57	2.1	52	-	-	-	-		.	.		2.9	0.7
Le Heron, 2018	Effort based decision making task	PD	39	67.8	7.6	19.04	.	.	29.5	9.9	ACE	91.1	7.9	BDI	14.3	7.7
		HC	32	68.9	6.9	40.6	-	-	-	-		95.6	3.8		3.8	3.7
Mcguigan, 2018	Cognitive effort task	PD	20	67.1	9.1	40	5.4	4.9	27.3	18.1	MoCA	27.7	1.92	BDI	9.5	4.98
		HC	20	61.1	13.6	40	-	-	-	-		28.1	1.37		3.55	4.13
Torta, 2009	Cambridge gamble task	PD	15	58.4	6.9	13.33	13.2	3.2	16.52	7.5	MMSE	28.2	1.5	BDI	10	6.6
		HC	13	58.2	5.7	15.38	-	-	-	-		.	.		.	.
Sharp, 2013	Vancouver gambling task (modified)	PD	18	65.47	9.17	27.78	5.59	4.04	20.65	6.91	MOCA	27.94	1.14	BDI	6.06	3.78
		HC	18	66.76	5.83	50	-	-	-			28.85	1.32		5.18	3.57
**Option valuation in Parkinson's with psychiatric syndrome vs without**				**Age (years)**			**Disease duration (years)**		**UPDRS**		**Cognition**			**Depression**		
**Study**	**Task**	**Group**	**n**	**Mean**	**SD**	**% female**	**Mean**	**SD**	**Mean**	**SD**	**Cognitive measure**	**Mean**	**SD**	**Depression measure**	**Mean**	**SD**
Haagensen, 2020	Game of dice task	PD	13	61.4	9.7	46.15	4.5	2	22.8	6.9	MoCA	28.7	1.3	BDI	7	5
		ICD	13	59.4	10.9	38.46	6.5	3.6	22.6	6		28.7	0.9		7.4	6.5
Kobayashi, 2019	Economic choice task	PD	15	63	6.58	40	6.93	6.2	39	14.6	MMSE	28.3	1.76	.	.	.
		ICD	10	63.1	9.68	40	8.4	2.99	49.9	26.3		28.3	1.77	.	.	.
Le Heron, 2018	Effort based decision	PD	18	68.2	6.5	44.44	.	.	27.1	13.2	ACE	93.5	5	BDI	11	7
		Apathy	21	67.5	8.5	19.05	.	.	29.5	9.9		89.4	9.4		17.1	7.4

Voon, 2011	Gambling task	PD	14	54.5	12.5	71.4	.	.	.	.	.	.	.	.	.	.
		ICD	14	51.5	8.3	71.4	.	.	.	.	.	.	.	.	.	.

PD = Parkinson's disease, HC = healthy controls, ICD = Impulse control disorder, SD = standard deviation, IQR = interquartile range, '.' = not reported, '-' = not applicable, UPDRS = Unified Parkinson's Disease Rating Scale, MMSE = Mini-mental state examination, MoCA = Montreal cognitive assessment, ACE = Addenbrooke's cognitive examination, BDI = Beck depression inventory, DASS = Depression, Anxiety and Stress Scale, GDS = Geriatric depression scale, MADRS = Montgomery-Asberg Depression Rating Scale

Supplement table 2. Reinforcement Learning study characteristics and participant demographics.

Reinforcement learning in Parkinson's vs Healthy Controls				Age (years)			Disease duration (years)		UPDRS		Cognition			Depression		
Study	Task	Group	n	Mean	SD or (IQR)	% female	Mean	SD or (IQR)	Mean	SD or (IQR)	Measure	Mean	SD or (IQR)	Measure	Mean	SD or (IQR)
Castrioto, 2015	IGT	PD	20	53.2	6.6	45	10.3	3.8	12.3	6.1	MDRS	137.6	4.2	.	.	.
		HC	24	54.9	7.6	62.5	-	-	-	-		140.7	2.4		.	.
Balusubramani, 2015	Probabilistic rewarded categorisation learning task	PD	30	.	.	18.75	9.5	.	.	.	MMSE	.	.	BDI	.	.
		HC	20	.	.	13.04	-	-	-	-					.	.
Bodi, 2009	Probabilistic classification task	PD	22	44.8	5.2	30.77	0.266	0.166	27.5	6.1	.	.	.	HAM-D	4.2	1.4
		HC	20	45.3	8.5	25	-	-	-	-		.	.		.	.
Buelow, 2014	IGT	PD	24	68.04	7.86	45.8	.	.	29.89	13.14	MMSE	28.33	1.63	GDS	2.5	1.91
		HC	14	69.62	6.36	53.85	-	-	-	-		29.31	1.11		1.15	1.57
Cavanagh, 2017	Cost of conflict task	PD	28	69.75	8.59	39.28	5.54	4.18			MMSE	28.64	1.06	BDI	7.64	5.23
		HC	28	69.21	9.23	39.28	-	-	-	-		28.82	1.02		4.93	4.69
Czernecki, 2002	IGT	PD	23	57.6	2.1	60.87	14.9	1.2	12.4	2	MDRS	139.1	0.8	MADRS	8.3	1.4
		HC	28	58.1	1.7	35.71	-	-	-	-		141.1	0.3		6.2	0.8
Delazer, 2009	IGT	PD	20	68.5	5.9	25	5.25	6.38	17.6	8.7	MMSE	27.8	1.9	HADS	6.6	3.2
		HC	20	71.3	3.5	85	-	-	-	-		29.8	0.4		.	.
Euteneuer, 2009	IGT	PD	21	67.6	7.31	67	7.14	6.06	17.7	9.2	MMSE	29	1.1	BDI	3.9	2.12
		HC	23	64.4	8.56	48						29.65	0.65		0.83	1.3
Evens, 2015	IGT	PD	32	65.12	8.25	25	7.38	4.33	16.58	7.36	MMSE	29.25	0.98	MADRS	4.33	3.68
		HC	32	65.53	5.94	31	-	-	-	-		29.34	0.83		1.59	2.19
Garofalo, 2017	Instrumental conditioning task	PD	17	63.29	9.94	50	16.42	28.77	.	.	MMSE	28.41	1.37	BDI	12.66	7.83
		HC	24	61.91	5.83	44	-	-	-	-		28.94	1.54		8.88	4.94
Gescheidt, 2012	IGT	PD	19 (early onset)	50.32	8.74	25	11.32	6.42	14.6	8.7	MMSE	29.37	0.96	MADRS	.	.
		HC	20	49.95	9.03	26.3	-	-	-	-		29.7	0.47		.	.
Graef, 2010		PD	15	65.27	8.14	40	4.3	4	19.64	7.7	MMSE	29	1	BDI	6.93	5.55

	Probabilistic reversal learning task	HC	16	67.75	4.55	37.5	-	-	-	-		28.64	1.15		5.31	2.98
Herzallah, 2017	Rewarded categorisation task	PD	17	59.4	12.6	11.76	5.24	4	28.6	14.6	MMSE	28.5	1.1	BDI	8.3	5.2
		HC	15	54.3	12.3	33.33	-	-	-	-		29.3	0.7		6.7	5.3
Housden, 2010	Rewarded salience attribution test	PD	18	67.7	5.5	38.89	12.9	8.3	20	6.6	MMSE	28.6	2.1	BDI	12.9	9.9
		HC	20	65.5	6	33.33	-	-	-	-		29.4	0.8		11.3	6.9
Ibarretxe-Bilbao, 2009	IGT	PD	24	56.13	8.5	33	3.06	1.6	14.67	3.5	MMSE	29.63	0.5	BDI	.	.
		HC	24	57.58	8.9	33	-	-	-	-		29.83	0.4			
Kobayakawa, 2008	IGT	PD	34	69.9	8.9	64.7	6.4	3.4	.	.	MMSE	28	2.2	SDS	36.1	8.9
		HC	22	67.6	6.9	40.9	-	-	-	-		28.8	1.6		29.1	6
Kobayakawa, 2010	IGT	PD	14	68.9	8	50	5.6	2.7	.	.	MMSE	28.2	1.9	SDS	32.9	7.8
		HC	22	67.6	6.9	69.2	-	-	-	-		28.8	1.6		29.1	6
Mapelli, 2014	IGT	PD	15	61.4	9.6	25	4.8	3.4	8.9	4	MMSE	28.3	1.2	BDI	Excluded if >14	
		HC	15	60.7	9.8	26.7	-	-	-	-		27.86	1.5		.	.
Mimura, 2006	IGT	PD	18	68.9	7	72.2	.	.	.	.	MMSE	27.8	1.9	ZSRD	39.4	6.9
		HC	20	.	.	70	-	-	-	-		29.1	1.5		30.5	6
Pagonabarraga, 2007	IGT	PD	35	67.2	8	37.2	8.4	5	21.2	8	MDRS	133	6	.	.	.
		HC	31	70.2	10	47.7	-	-	-	-		136	5		.	.
Paz-Alonso, 2019	IGT	PD	17	61	8.7	11.8	7	(4-10)	25.9	8.2	.	.	.	HADS-D	2.2	2.6
		HC	18	63	9.7	16.7	-	-	-	-		.	.		1.9	1.3
Perretta, 2005	IGT	PD (late stage)	16	77.7	6	50	.	.	27.2	1.3	MMSE	Excluded if <27	.	BDI	12.7	1.5
		PD (early stage)	16	72.4	2.3	43.75	.	.	11.3	1.1					6.9	0.7
		HC	19	72.6	8.28	42.1	-	-	-	-					5.2	0.8
Pignatti, 2012	IGT	PD	15	64.27	10.5	.	.	.	.	.	MMSE	28.31	1.11	.	.	.
		HC	16	41.56	13.9	.	-	-	-	-					.	.
Piray, 2014	Rewarded categorisation task	PD	40	63.33	3.98	23.07	9.72	2.64	19.6	6.42	MMSE	27	0.93	BDI	8	1.69
		HC	20	66.45	4.7	35	-	-	-	-		27.65	1.18		7.75	1.97
Poletti, 2010	IGT	PD (off)	24	64.9	5.8	27	0 (de novo)		14.3	8.1	MMSE	28.8	2	GDS	4.7	3.3
		HC	25	65.4	2.2	44						28.5	1.8		2.8	1.4
Thiel, 2003	IGT	PD	5	62.6	12.5	40	8	2.55	20.6	6.47	MMSE	.28.4	2.07	.	.	.
		HC	5	44.2	21.3	20	-	-	-	-						
Xi, 2015	IGT	PD	15	60.73	11.7	53	4.33	5.05	15.87	8.96	MMSE	27.6	4.63	HAMD	4.67	1.29

		HC	15	56.33	14.5	60	-	-	-	-		29.4	1.12		3.8	1.37
Yildirim, 2020	IGT	PD	39	65.9	7.3	21	7.8	4.3	11.6	6.1	MMSE	.	.	.	.	.
		HC	37	64.4	8	24	-	-	-	-		.	.		.	.

Reinforcement learning in Parkinson's with psychiatric syndrome vs without				Age			Disease duration (years)		UPDRS		Cognition				Depression	
Study	Task	Group	n	Mean	SD or (IQR)	% female	Mean	SD or (IQR)	Mean	SD or (IQR)	Measure	Mean	SD or (IQR)	Depression measure	Mean	SD or (IQR)
Balconi, 2018	IGT	PD	20	63.9	7.1	15	.	.	13	9.3	.	.	.	BDI	11.3	6
		PG	17	60.7	6.1	17.65	.	.	17	7.8		.	.		15.9	9.1
Balusubramani, 2015	Rewarded categorisation task	PD	14	.	.	21.4	8.35	.	.	.	MMSE	.	.	BDI	.	.
		ICD	16	.	.	12.5	9.56	.								
Bentivoglio, 2012	IGT	PD	17	63.94	9.2	35.29	7.3	4.4	22.5	6.9	MMSE	28.6	1.4	HAM-D	5.6	4.1
		ICD	17	62	10.1	17.65	6.9	3.8	23.8	11		28.4	1.6		7.5	6.3
Biars, 2019	IGT	PD	24	60.5	7.8	12.5	11.9	7.1	37.2	14.8	DRS	136.5	5.9	BDI	8.5	5.3
		ICD	24	61.2	8.3	12.5	13.2	7.1	21.6	9.9		138	3.5		11.2	6.8
Buelow, 2014	IGT	PD	14	69	6.19	57.14	.	.	29.62	6.85	MMSE	28.71	1.38	GDS	2.14	1.41
		Apathy	10	66.7	9.96	30	.	.	29.89	13.14		27.8	1.87		3	2.45
Garofalo, 2017	Instrumental conditioning task	PD	17	63.29	9.94	44	10.94	10.38	.	.	MMSE	28.94	1.54	BDI	8.88	4.94
		Psychosis	12	60.83	6.6	50	16.42	28.77	.	.		28.41	1.37		12.66	7.83
Herzallah, 2017	Rewarded categorisation task	PD	17	59.4	12.6	11.76	5.24	4	.	.	MMSE	28.5	1.1	BDI	8.3	5.2
		MDD	13	55.2	11.9	69.2	4.84	3.7	28.6	14.6		27.5	1.8		26.9	7.7
Housden, 2010	Rewarded salience attribution test	PD	18	67.7	5.5	33.33	12.9	8.3	21.3	0.4	MMSE	29.4	0.8	BDI	11.3	6.9
		ICD	18	62.3	7.6	38.89	13.9	9	20	6.6		28.6	2.1		12.9	9.9
Martinez-Horta, 2013	IGT	PD	17	65.06	4.85	.	5.38	4.25	18	4.61	MMSE	28.94	1.24	HADS	3.88	3.6
		Apathy	17	68.25	6.06	.	6.75	4.93	18.65	5.8		28.55	1.23		5.65	2.92
Paz-Alonso, 2019	IGT	PD	17	61	8.7	11.8	7	(4-10)	25.9	8.2	.	.	.	HADS	2.2	2.6
		ICD	18	62.3	7.6	11.1	8	(5.1-10)	22.31	6.6		.	.		3.1	2.4
Pineau, 2016	IGT (adapted)	PD	20	55	(40-62)	35	5.5	(4-12)	8.5	(0-34)	MDRS	139	(131-143)	.	.	
		ICD	17	55	(37-65)	17.64	7	(2-10)	7	(0-23)		140	(133-144)		.	.
Piray, 2014	Rewarded categorisation task	PD	40	63.33	3.98	25.9	8.87	3.14	19.6	6.42	MMSE	27	0.93	BDI	8	1.69
		ICD	16	64.38	3.32	11.1	9.63	2.45	19	5.32		27.19	1.11		6.75	1.69
Poletti, 2011	IGT	PD	12	63.92	7.17	16.66	.	.	.	.	MMSE	28.72	2.41	GDS-15	3.33	2.34
		Alexithymia	12	66.17	5.2	41.66	.	.	.	.		28.49	1.94		6.33	3.89
Rossi, 2010	IGT	PD	13	65.1	3.8	23	.	.	14.7	6.7	MMSE	.	.	MADRS	14.1	7.9

		PG	7	61.4	6.9	14	.	.	17	9.1		Excluded if <24	.		17.1	6.5
Sáez-Francàs, 2014	IGT	PD	56	62.64	9.06	32.14	4.31	3.65	18.41	5.82	MMSE	.	.	HAM-D	3.64	3.37
		Fatigue	33	61.73	9.85	39.4	4.94	3.44	20.85	6.31		Excluded if <26	.		8.64	7.06
Timmer, 2017	Rewarded task-switching paradigm	PD	22	61.1	7.6	36.4	.	.	21.9	6.8	MMSE	28.6	1.2	BDI	4.3	2.3
		MDD	19	58.4	5.3	36.8	.	.	.	.		28.5	1.3		8.7	5

PD = Parkinson's disease, HC = healthy controls, IGT = Iowa Gambling Task, ICD = Impulse control disorder, MDD = Major depressive disorder, SD = standard deviation, IQR = interquartile range, '.' = not reported, '-' = not applicable, UPDRS = Unified Parkinson's Disease Rating Scale, MMSE = Mini-mental state examination, MoCA = Montreal cognitive assessment, MDRS = Mattis dementia rating scale, BDI = Beck depression inventory, HAM-D = Hamilton depression rating scale, HADS = Hospital anxiety and depression scale ZSRD = Zung Self-Rating Depression scale, DASS = Depression, Anxiety and Stress Scale, GDS = Geriatric depression scale, MADRS = Montgomery-Asberg Depression Rating Scale, SDS = Self-rated depression scale

Supplement table 3. Response Vigor and Reward Bias study characteristics and participant demographics.

Response vigor in Parkinson's vs Healthy Controls				Age			Disease duration		UPDRS		Cognition			Depression		
Study	Task	Group	n	Mean	SD	% female	Mean	SD	Mean	SD	Cognitive measure	Mean	SD	Depression measure	Mean	SD
Muhammed, 2016	Speed of gaze shifting task	PD	16	67.3	6.4	.	3.2	2.2	19.4	9.8	MoCA	27.8	2.3	BDI	11.7	5.5
		HC	31	65.9	5.6	.	-	-	-	-		28.2	1.6		15.2	7.8
Renfroe, 2016	Rewarded speed of response to specific visual stimuli	PD	18	66	7.99	22.2	8.39	4.57	26	9.55	.	.	.	BDI	7.82	4.85
		HC	15	70	6.94	40	-	-	-	-		.	.		1.8	2.44
Timmer, 2018	Stroop-like incentive task switching	PD	23	61	7.4	39.13	4.5	2.2	21.8	6.7	MMSE	28.5	1.3	BDI	4.1	2.3
		HC	23	60.9	5.9	39.13	-	-	-	-		28.8	1.2		3.1	2.1
**Response vigor in Parkinson's with psychiatric syndrome vs without**				Age			Disease duration		UPDRS		Cognition			Depression		
Study	Task	Group	n	Mean	SD	% female	Mean	SD	Mean	SD	Cognitive measure	Mean	SD	Depression measure	Mean	SD
Drew, 2020	Speed of gaze shifting task	PD	26	67.19	5.92	26.9	4.87	4.09	18.62	9.38	MoCA	27.77	1.95	BDI	13	7.1
		ICD	23	63.7	7.56	47.82	8.71	4.25	24.22	16.94		27.39	2.55		12.26	5.84
Evans, 2010	Card arranging reward responsivity test	PD	20	59.5	7.9	.	13.6	8	19.2	2.5	MMSE	29.1	(27-30)	GDS	9	4.9
		DDS	20	55.4	7.6	.	14	5.7	22.7	2.5		29	(24-30)		17.6	6.4
Lawrence, 2011	Spatial search task	PD	10	59.6	6.4	.	.	.	25.6	11	MMPD	25.6	11	GDS	4.7	2
		Apathy	10	61.7	6.1	.	.	.	28.2	11.4		29.3	2.8		8.2	3.5
Muhammed, 2016	Speed of gaze shifting task	PD	16	65.9	5.6	.	6.8	4.6	18.4	2.6	MoCA	28.2	1.6	BDI	11.7	5.5
		Apathy	14	67.3	6.4	.	3.2	2.2	20.4	2.4		27.8	2.3		15.2	7.8
Timmer, 2018	Stroop-like incentive task switching	PD	23	61	7.4	39.13	4.5	2.2	21.8	6.7	MMSE	28.5	1.3	BDI	4.1	2.3
		Depression	22	58.4	5.7	36.36	5	3.5	23.1	9.6		28.4	1.4		9.6	6.1

PD = Parkinson's disease, HC = healthy controls, ICD = Impulse control disorder, SD = standard deviation, IQR = interquartile range, '.' = not reported, '-' = not applicable, UPDRS = Unified Parkinson's Disease Rating Scale, MMSE = Mini-mental state examination, MoCA = Montreal cognitive assessment, MMPD = Mini-mental Parkinson's examination BDI = Beck depression inventory, GDS = Geriatric depression scale

Supplement table 4. Study summary statistics, tasks and measures included in the Meta-Analysis of Parkinson's versus healthy controls.

Reward processing summary statistics for Parkinson's disease patients versus healthy controls													Tasks and Measures Included in the Meta-Analysis.	
Author, Year	Category	OFF/ON	N (HC)	N (PD)	t	F	M (HC)	SD (HC)	M (PD)	SD (PD)	d	Vard	Task	Measure
Bayard et al, 2016 (ON)	OV	ON	96	78			6.90	7.65	2.77	8.41	0.52	0.02	Game of dice task	Net score
Chong et al, 2015 (ON)	OV	ON	26	26							-0.35	0.08	Effort based decision making task (apple gathering)	Effort indifference point (mean effect size across stake levels, fig 4)
Chong et al, 2015 (OFF)	OV	OFF	26	26		2.70					0.46	0.08	Effort based decision making task (apple gathering)	Effort indifference point
Brandt et al, 2015 (ON)	OV	ON	15	15			14.93	15.72	-8.67	17.67	1.41	0.17	Game of dice task	Net score (fig 1)
Kobayashi et al, 2019 (ON)	OV	ON	21	15			0.21	0.78	0.30	0.66	0.12	0.11	Economic choice task	Relative risk aversion coefficient (fig 2C, first session)
Kobayashi et al, 2019 (OFF)	OV	OFF	21	15			0.21	0.78	0.97	0.70	1.02	0.13	Economic choice task	Relative risk aversion coefficient (fig 2C, first session)
Le Heron et al, 2018 (ON)	OV	ON	32	18	1.30						0.38	0.09	Effort based decision making task (apple gathering)	Mean difference in proportion of offers accepted
Mcguigan et al, 2018 (ON)	OV	ON	20	20	-0.32						-0.10	0.10	Cognitive effort task	Mean difference in k-value
Mcguigan et al, 2018 (OFF)	OV	OFF	20	20	2.79						0.88	0.11	Cognitive effort task	Mean difference in k-value
Sharp et al, 2013 (ON)	OV	ON	18	18	0.43						0.14	0.07	Vancouver gambling task	Gain phase adjust y intercept
Sharp et al, 2013 (OFF)	OV	OFF	18	18	1.79						0.60	0.07	Vancouver gambling task	Gain phase adjust y intercept
Torta et al, 2009 (ON)	OV	ON	13	15							0.24	0.14	Cambridge gamble task	Average bet (across risk levels)
Torta et al, 2009 (OFF)	OV	OFF	13	15							0.23	0.14	Cambridge gamble task	Average bet (across risk levels)
Cools et al, 2003 (ON)	OV	ON	12	12			54.91	12.09	59.65	16.11	-0.33	0.17	Incentivised decision making task	Mean % bets across ascending & descending conditions
Cools et al, 2003 (OFF)	OV	OFF	12	12			54.91	12.09	58.95	15.73	-0.29	0.17	Incentivised decision making task	Mean % bets across ascending & descending conditions
Le Bouc et al, 2016 (ON)	OV	ON	25	24			80.89	7.75	78.56	12.49	0.23	0.08	Effort based decision making task: Binary choice task	Choice of effort level at the highest stake level. (fig 4D)
Le Bouc et al, 2016 (OFF)	OV	OFF	25	24	3.51						1.00	0.09	Effort based decision making task: Binary choice task	Choice of effort level at the highest stake level. (fig 4D)
Castrioto et al, 2015 (ON)	RL	ON	24	20			4.38	40.22	-1.79	44.72	0.15	0.09	Iowa Gambling Task	Baseline final round mean score. (fig 1A)
Castrioto et al, 2015 (OFF)	RL	OFF	24	20			4.38	40.22	0.98	40.34	0.08	0.09	Iowa Gambling Task	Baseline final round mean score. (fig 1A)
Balusubramani et al, 2015 (ON)	RL	ON	20	14			64.10	84.84	61.54	78.69	0.03	0.12	Probabilistic rewarded categorisation learning task	% Optimality during expected reward (fig 3A)
Balusubramani et al, 2015 (OFF)	RL	OFF	20	26			64.10	84.84	43.07	99.38	0.23	0.09	Probabilistic rewarded categorisation learning task	% Optimality during expected reward (fig 3A)
Bodi et al, 2009 (ON)	RL	Medicated	20	22			81.18	18.43	89.18	17.12	-0.45	0.10	Feedback-based probabilistic classification task	Final block % optimal decisions (fig 2)
Bodi et al, 2009 (Never medicated)	RL	Never medicated	20	26			81.18	18.43	59.53	18.61	1.17	0.10	Feedback-based probabilistic classification task	Final block % optimal decisions (fig 2)
Buelow et al, 2014 (ON)	RL	ON	14	24							1.38	0.14	Iowa Gambling Task	Final block score (authors provided)

Piray et al, 2014 (ON)	RL	ON	20	15			0.12	0.16	0.14	0.20	-0.10	0.12	Probabilistic learning task	Actor's learning rate (fig 6C)
Piray et al, 2014 (OFF)	RL	OFF	20	25			0.12	0.16	0.08	0.13	0.29	0.09	Probabilistic learning task	Actor's learning rate (fig 6C)
Yildirim et al, 2020 (ON)	RL	ON	37	39			0.65	5.72	-0.11	5.77	0.13	0.05	Iowa Gambling Task	Final block mean score (author provided)
Gescheidt et al, 2012 (ON)	RL	ON	20	19			10.30	29.42	-6.00	25.26	0.59	0.11	Iowa Gambling Task	Mean total score
Herzallah et al, 2017 (ON)	RL	ON	15	17			76.49	17.36	72.10	16.62	0.26	0.13	Feedback-based probabilistic classification task	Mean % optimal responses for positive feedback (fig 5A)
Housden et al, 2010 (ON)	RL	ON	20	18			56.50	22.40	27.00	19.10	1.41	0.13	Salience Attribution Test	Visual analogue scale rating for high probability stimuli
Mapelli et al, 2014 (ON)	RL	ON	15	15			9.81	10.19	4.74	5.11	0.63	0.14	Iowa Gambling Task	Final block mean score (fig 1)
Mimura et al, 2006 (ON)	RL	ON	20	18			3.70	7.35	-0.33	6.59	0.58	0.11	Iowa Gambling Task	Final 50 cards mean score
Pagonabarraga et al, 2007 (ON)	RL	ON	31	35			6.10	17.00	-10.80	19.00	0.93	0.07	Iowa Gambling Task	Mean total score
Paz-Alonso et al, 2019 (ON)	RL	ON	18	17			40.60	18.82	41.80	15.38	-0.07	0.11	Iowa Gambling Task	Mean difference % optimal choice, final block. (supplement fig 1)
Garofalo et al, 2017 (ON)	RL	ON	24	17			21.50	5.26	15.15	6.68	1.08	0.11	Instrumental conditioning task	Reward learning index (fig 2B)
Czernecki et al, 2002 (ON)	RL	ON	28	23			8.32	11.06	2.86	16.59	0.40	0.08	Iowa Gambling Task	Final block mean score (fig 1, second session)
Czernecki et al, 2002 (OFF)	RL	OFF	28	23			8.32	11.06	5.36	10.89	0.27	0.08	Iowa Gambling Task	Final block mean score (fig 1, second session)
Graef et al, 2010 (ON)	RL	ON	15	14	1.80		68.49	14.07	60.07	11.08	0.67	0.15	Instrumental learning task	% correct choices with constant reward contingencies
Graef et al, 2010 (OFF)	RL	OFF	15	14	2.19		68.49	14.07	59.05	8.86	0.81	0.15	Instrumental learning task	% correct choices with constant reward contingencies
Thiel et al, 2003 (OFF)	RL	OFF	5	5			53.6	15.8	61.2	17.1	-0.46	0.41	Iowa Gambling Task	Mean number of advantageous cards selected.
Perretta et al, 2005 (late PD)	RL	ON late PD	19	16			7.80	1.31	6.80	1.60	0.69	0.12	Iowa Gambling Task	Mean total score
Perretta et al, 2005 (early PD)	RL	ON early PD	19	16			7.80	1.31	7.60	1.60	0.14	0.12	Iowa Gambling Task	Mean total score
Kobayakawa et al, 2008 (ON)	RL	ON	22	34		4.80	4.90	12.20	-16.00	21.57	0.60	0.08	Iowa Gambling Task	Group difference in choice patterns (advantageous - disadvantageous)
Kobayakawa et al, 2010 (ON)	RL	ON	22	14		2.96					0.59	0.12	Iowa Gambling Task	Group difference in choice patterns (advantageous - disadvantageous)
Poletti et al, 2010 (OFF)	RL	OFF (de novo)	25	30			6.00	6.82	3.07	12.07	0.29	0.07	Iowa Gambling Task	Mean total score
Delazer et al, 2009 (ON)	RL	ON	20	20		8.81					0.94	0.11	Iowa Gambling Task	Group difference in choice patterns (advantageous - disadvantageous)
Euteneuer et al, 2009 (ON)	RL	ON	23	21		0.65					0.24	0.09	Iowa Gambling Task	Group difference in choice patterns (advantageous - disadvantageous)
Ibarretxe-Bilbao et al, 2009 (ON)	RL	ON	24	24		14.56					1.10	0.10	Iowa Gambling Task	Group difference in choice patterns (advantageous - disadvantageous)
Pignatti et al, 2012 (ON)	RL	ON	16	15			12.50	14.87	7.47	16.06	0.33	0.13	Iowa Gambling Task	Total score over the last 50 choices
Evens et al, 2015 (ON)	RL	ON	32	32			-7.31	20.78	-2.09	16.27	-0.28	0.06	Iowa Gambling Task	Mean total score

Xi et al, 2015 (ON)	RL	ON	15	15		10.67					1.19	0.16	Iowa Gambling Task	Group difference in choice patterns (advantageous - disadvantageous)
Cavanagh et al, 2017 (ON)	RL	ON	28	28	-1.64						-0.44	0.07	Cost of conflict task	% selection of most (A) vs least (D) rewarding stimuli
Cavanagh et al, 2017 (OFF)	RL	OFF	28	28	-1.44						-0.38	0.07	Cost of conflict task	% selection of most (A) vs least (D) rewarding stimuli
Muhammed et al, 2016 (ON)	RV	ON	31	30	5.50						0.60	0.07	Rewarded speed of gaze shifting task	Reward sensitivity in peak velocity
Timmer et al, 2018 (ON)	RV	ON	23	23			5.00	4.50	14.40	4.40	-2.11	0.14	Rewarded task switching paradigm	Reward related speeding 'repeat' condition
Timmer et al, 2018 (OFF)	RV	OFF	23	23			5.00	4.50	3.80	4.70	0.26	0.09	Rewarded task switching paradigm	Reward related speeding 'repeat' condition

Supplement table 5. Study summary statistics, tasks and measures included in the Meta-Analysis of Parkinson’s with & without psychiatric syndrome

Reward processing summary statistics for Parkinson's disease versus Parkinson's disease plus psychiatric syndrome													Tasks and Measures Included in the Meta-Analysis.	
Author, Year	Category	Psychiatric syndrome	N (PD)	N (PD+PSYCH)	t	F	M (PD)	SD (PD)	M (PD +Psych)	SD (PD+Psych)	d	Vard	Task	Measure
Balusubramani et al, 2015 (ON)	RL	ICD	14	16			61.54	78.69	78.97	61.56	-0.25	0.13	Probabilistic reward and punishment learning task	% Optimality during expected reward (fig 3A)
Piray et al, 2014 (ON)	RL	ICD	15	16			0.135	0.198	0.067	0.06	0.47	0.13	Probabilistic learning task	Actor's learning rate (fig 6C)
Housden et al, 2010 (ON)	RL	ICD	18	18			27	19.1	42.7	19.7	-0.81	0.12	Salience Attribution Test	Visual analogue scale rating for high probability stimuli
Biars et al, 2019 (ON)	RL	ICD	24	24			3.5	9.7	2.75	8.2	0.08	0.08	Iowa Gambling Task	Final block score (authors provided)
Balconi et al, 2018 (ON)	RL	PG	20	17		7.60					0.91	0.12	Iowa Gambling Task	Group difference in choice patterns (advantageous - disadvantageous)
Paz-Alonso et al, 2019 (ON)	RL	ICD	17	18			41.80	15.38	27.00	13.94	1.01	0.13	Iowa Gambling Task	Mean difference % optimal choice, final block. (supplement fig 1)
Pineau et al, 2016 (ON)	RL	ICD	20	17			15.00	15.56	14.00	9.63	0.08	0.11	Iowa Gambling Task	Mean number of cards selected from winning deck
Bentivoglio et al, 2012 (ON)	RL	ICD	17	17			8.40	22.10	-4.60	33.10	0.46	0.12	Iowa Gambling Task	Mean total score
Rossi et al, 2010 (ON)	RL	PG	13	7	2.60						1.22	0.26	Iowa Gambling Task	Final block mean net score
Garofalo et al, 2017 (ON)	RL	Psychosis	17	12			15.15	6.68	8.67	8.34	0.88	0.16	Instrumental conditioning task	Reward learning index (fig 2B)
Sáez-Francàs et al, 2014 (ON)	RL	Fatigue	56	33			1.79	14.80	-4.18	11.13	0.44	0.05	Iowa Gambling Task	Final three blocks mean net score
Martinez-Horta et al, 2013 (ON)	RL	Apathy	17	20			-1.82	5.80	4.10	4.80	-1.12	0.13	Iowa Gambling Task	Final block score
Buelow et al, 2014 (ON)	RL	Apathy	14	10			22.12	16.80	-24.00	14.87	2.88	0.34	Iowa Gambling Task	Final block score (authors provided)
Herzallah et al, 2017 (ON)	RL	MDD	17	13			72.10	16.62	49.30	22.14	1.19	0.16	Feedback-based probabilistic classification task	Mean % optimal responses for positive feedback (fig 5A)
Timmer et al, 2017 (ON)	RL	MDD	22	19			0.10	0.06	0.07	0.06	-0.50	0.10	Deterministic reversal learning paradigm	Error rate for expected reward

Poletti et al, 2011 (OFF)	RL	Alexithymia	12	12			4.17	8.37	3.00	6.35	0.16	0.17	Iowa Gambling Task	Final block score
Kobayashi et al, 2019 (ON)	OV	ICD	15	10			0.30	0.66	-0.19	0.17	-0.93	0.18	Economic choice task	Relative risk aversion coefficient (fig 2C, first session)
Le Heron et al, 2018 (ON)	OV	Apathy	18	21	2.33						0.75	0.11	Effort based decision making task (apple gathering)	Mean difference in proportion of offers accepted
Voon et al, 2011 (ON)	OV	ICD	14	14							-0.68	0.14	Gambling task	Proportion of risky choices in gain phase (across risk levels, fig 2A)
Timmer et al, 2018 (ON)	RV	MDD	23	22			14.40	4.40	17.70	3.90	-0.79	0.10	Rewarded task-switching paradigm	Reaction time reward benefit (repeat)
Evans et al, 2010 (ON)	RV	DDS	20	20		12.7					-1.13	0.12	Card arranging reward responsivity objective test	Reward responsivity
Drew et al, 2020 (ON)	RV	ICD	26	23		2.78					-0.48	0.08	Rewarded speed of gaze shifting task	Residual velocity reward sensitivity
Lawrence et al, 2011 (ON)	RV	Apathy	10	10		0.38					0.20	0.28	Rewarded spatial search task	Reward related speeding

Supplement table 6. Study summary statistics, tasks and measures included in the Meta-Analysis of Parkinson's on and off dopaminergic medication

Reward processing summary statistics for Parkinson's disease patients ON vs OFF dopaminergic medication							Tasks and Measures Included in the Meta-Analysis.	
Author, Year	Category	N	t	F	d	Vard	Task	Measure
Czernecki et al, 2002	RL	22		0.05	0.048	0.046	Iowa Gambling Task	Number of advantageous minus disadvantageous choices
Graef et al, 2010	RL	14	-0.374		-0.100	0.072	Instrumental learning task	% correct choices with constant reward contingencies
Cavanagh et al, 2017	RL	28		2.87	0.320	0.0375	Value of volition task	% selection of A (90% reward) vs C (70% reward) – B (10% reward) vs D (30% reward) stimuli
Bodi et al, 2009	RL	26		13.47	0.720	0.0385	Feedback-based probabilistic classification task	Pattern of optimal decision selection over time
Chong et al, 2015	OV	26		25.9	0.998	0.058	Effort based decision making task (apple gathering)	Effort indifference point
Kobayashi et al, 2019	OV	24		7.48	0.558	0.048	Economic choice task	Relative risk aversion coefficient
Le Heron et al, 2018	OV	39	2.45		0.392	0.028	Effort based decision making task (apple gathering)	Mean difference in proportion of offers accepted
Mcguigan et al, 2018	OV	20	3.05		0.682	0.062	Cogntiive effort task	Mean difference in k-value
Cools et al, 2003	OV	12		5.9	0.701	0.104	Incentivised decision making task	Difference score of bets places in ascending and descending conditions.
Le Bouc et al, 2016	OV	20	2.76		0.617	0.060	Effort based decision making task: Binary choice task	Choice of effort level at the highest stake level.
Sharp et al, 2013	OV	18	1.17		0.276	0.058	Vancouver gambling task	Adjusted y intercept
Timmer et al, 2018	RV	23		3.031	0.363	0.046	Rewarded task switching paradigm	Reward related speeding 'repeat' condition
Muhammed et al, 2016	RV	30		10.8	0.600	0.039	Rewarded speed of gaze shifting task	Reward sensitivity in peak velocity
Evans et al, 2010	RV	38		0.4	0.103	0.026	Card arranging reward responsivity objective test	Reward responsivity
Drew, 2020	RV	26		5.178	0.446	0.042	Rewarded speed of gaze shifting task	Residual velocity reward sensitivity

Modified version of the Newcastle-Ottawa Scale for Assessing the Quality of Nonrandomized Studies in Meta-Analyses, used to assess potential sources of bias.

Potential sources of bias were assessed using a modified version of the Newcastle-Ottawa Scale for Assessing the Quality of Nonrandomized Studies in Meta-Analyses. The studies are scored on:

1. PD Definition: Is the case definition adequate?
A) Cases were defined as PD according to a validated assessment tool/criteria or by an experienced clinician
B) Cases were defined as PD according to a validated assessment tool/criteria but the method for assessing PD status was not stated.
C) Cases were described as 'clinically' but no further description was given.

2. PD Generality: Was a General sample of cases tested?
A) A General sample of PD was tested.
B) Recruitment of PD cases was restricted to a specific sub-sample (specific age range, hospitalised only etc.)

3. HC Selection: Selection of Controls
A) Controls were selected from the same population as cases
B) Controls were not selected from the same population as cases
C) No description

4. HC Definition: Definition of Controls
A) HC were defined clearly defined as having no current or past psychopathology
B) Controls were not clearly defined as having no current or past psychopathology.

Comparability (Comparability of cases and controls on the basis of the design or analysis)
1. Does the study control for Age: Yes/No/Unclear
2. Does the study control for Gender: Yes/No/Unclear
3. Does the study control for IQ: Yes/No/Unclear
4. Does the study control for Socioeconomic status: Yes/No/Unclear
5. Does the study control for PD severity: Yes/No/Unclear
6. Does the study control for medication status: Yes/No/Unclear

Key: A, B, C
Y = yes, N= no, N/A= Not applicable (such as if no healthy control group in study)

Supplement table 7. Quality rating of included studies

Study quality ratings			Modified version of the Newcastle-Ottawa Scale									
**Study**	**Year**	**Category**	**PD Definition**	**PD Generality**	**HC Selection**	**HC Definition**	**Age**	**Gender**	**IQ**	**Socioeconomic status**	**PD severity**	**PD medication**
Bayard[1]	2016	OV	A	A	B	A	Y	Y	N	N	Y	Y
Brandt[2]	2015	OV	A	A	C	B	Y	Y	Y	N	Y	Y
Chong[3]	2015	OV	B	A	A	A	Y	Y	N	N	Y	Y
Cools[4]	2003	OV	A	A	C	B	Y	N	Y	N	Y	Y
Haagensen[5]	2020	OV	C	A	A	B	Y	Y	N	N	Y	Y
Kobayashi[6]	2019	OV	A	A	C	B	Y	Y	N	N	Y	Y
Le Bouc[7]	2016	OV	C	B	A	B	Y	Y	N	N	Y	Y
Le Heron[8]	2018	OV	A	A	A	A	Y	Y	N	N	Y	Y
Mcguigan[9]	2018	OV	A	A	A	A	Y	Y	N	N	Y	Y
Torta[10]	2009	OV	A	B	C	B	Y	Y	N	N	Y	Y
Sharp[11]	2013	OV	A	A	C	B	Y	N	N	N	Y	Y
Voon[12]	2011	OV	A	A	n/a	n/a	Y	Y	N	N	Y	Y
Balconi[13]	2018	RL	A	A	n/a	n/a	Y	N	N	N	Y	Y
Balusubramani[14]	2015	RL	C	A	C	A	N	N	N	N	Y	Y
Bentivoglio[15]	2012	RL	A	A	n/a	n/a	Y	Y	Y	N	Y	Y
Biars[16]	2019	RL	C	B	n/a	n/a	Y	Y	Y	N	N	Y
Bodi[17]	2009	RL	C	B	C	A	Y	N	Y	Y	Y	Y
Buelow[18]	2014	RL	A	A	A	A	Y	Y	Y	N	Y	Y
Castrioto[19]	2015	RL	C	B	C	B	Y	Y	N	N	Y	Y
Cavanagh[20]	2017	RL	C	A	C	B	Y	Y	Y	N	Y	Y
Czernecki[21]	2002	RL	A	B	B	A	Y	Y	N	N	Y	Y
Delazer[22]	2009	RL	A	A	C	A	Y	Y	N	N	Y	Y
Euteneuer[23]	2009	RL	C	A	C	B	Y	Y	N	N	Y	Y
Evens[24]	2015	RL	A	A	A	A	Y	Y	N	N	Y	Y
Garofalo[25]	2017	RL	A	A	C	B	Y	Y	Y	N	Y	Y
Gescheidt[26]	2012	RL	A	A	C	A	Y	Y	N	N	Y	Y
Graef[27]	2010	RL	A	B	B	A	Y	Y	N	N	Y	Y
Herzallah[28]	2017	RL & RV	B	A	A	A	Y	N	N	N	Y	N
Housden[29]	2010	RL	C	A	A	A	Y	Y	Y	N	Y	Y
Ibarretxe-Bilbao[30]	2009	RL	A	A	A	B	Y	Y	N	N	Y	Y
Kobayakawa[31]	2008	RL	C	A	A	A	Y	Y	N	N	Y	Y
Kobayakawa[32]	2010	RL	C	A	A	A	Y	Y	N	N	Y	Y
Mapelli[33]	2014	RL	A	A	A	A	Y	Y	N	N	Y	Y
Martinez-Horta[34]	2013	RL	A	A	n/a	n/a	Y	N	N	N	Y	Y
Mimura[35]	2006	RL	C	A	B	A	Y	Y	N	N	Y	Y
Pagonabarraga[36]	2007	RL	A	A	A	A	Y	Y	N	N	Y	Y
Paz-Alonso[37]	2019	RL	A	A	A	B	Y	Y	Y	N	Y	Y
Perretta[38]	2005	RL	A	A	A	A	Y	Y	N	N	Y	Y
Pignatti[39]	2012	RL	A	B	C	B	N	N	N	N	N	N
Pineau[40]	2016	RL	A	A	n/a	n/a	Y	Y	N	N	Y	Y
Piray[41]	2014	RL	C	B	C	A	Y	N	Y	N	Y	N
Poletti[42]	2010	RL	A	A	C	B	Y	Y	N	N	Y	Y
Poletti[43]	2011	RL	A	B	n/a	n/a	Y	Y	N	N	N	Y
Rossi[44]	2010	RL	A	A	n/a	n/a	Y	Y	N	N	Y	Y
Sáez-Francàs[45]	2014	RL	C	A	n/a	n/a	Y	Y	N	N	Y	N
Thiel[46]	2003	RL	C	A	C	A	Y	N	N	N	Y	Y
Timmer[47]	2017	RL	A	A	A	A	Y	Y	Y	N	Y	Y
Xi[48]	2015	RL	A	B	C	B	Y	Y	N	N	Y	Y

Yildirim[49]	2020	RL	A	A	B	A	Y	Y	N	N	Y	Y
Drew[50]	2020	RV	A	B	C	A	Y	Y	N	N	Y	Y
Evans[51]	2010	RV	A	A	B	B	Y	Y	N	N	Y	Y
Lawrence[52]	2011	RV	A	A	n/a	n/a	Y	N	N	N	Y	Y
Muhammed[53]	2016	RV	C	A	B	B	Y	Y	Y	N	Y	N
Renfroe[54]	2016	RV	B	B	A	A	Y	Y	N	N	Y	Y
Timmer[55]	2018	RV	A	A	A	B	Y	N	Y	N	Y	Y

References

1. Bayard, S. *et al.* A multidimensional approach to impulsivity in Parkinson's disease: measurement and structural invariance of the UPPS Impulsive Behaviour Scale. *Psychol. Med.* **46**, 2931–2941 (2016).
2. J., B. *et al.* Betting on DBS: Effects of subthalamic nucleus deep brain stimulation on risk taking and decision making in patients with Parkinson's disease. *Neuropsychology* **29**, 622–631 (2015).
3. Chong, T. T. J. *et al.* Dopamine enhances willingness to exert effort for reward in Parkinson's disease. *Cortex* **69**, 40–46 (2015).
4. Cools, R., Barker, R. A., Sahakian, B. J. & Robbins, T. W. L-Dopa medication remediates cognitive inflexibility, but increases impulsivity in patients with Parkinson's disease. *Neuropsychologia* **41**, 1431–1441 (2003).
5. B.N., H. *et al.* Linking brain activity during sequential gambling to impulse control in Parkinson's disease. *NeuroImage. Clin.* **27**, 102330 (2020).
6. Kobayashi, S. *et al.* Dopaminergic influences on risk preferences of Parkinson's disease patients. *Cogn. Affect. Behav. Neurosci.* **19**, 88–97 (2019).
7. Le Bouc, R. *et al.* Computational Dissection of Dopamine Motor and Motivational Functions in Humans. *J. Neurosci.* **36**, 6623–6633 (2016).
8. Le Heron, C. *et al.* Distinct effects of apathy and dopamine on effort-based decision-making in Parkinson's disease. *Brain* **141**, 1455–1469 (2018).
9. S., M. *et al.* Dopamine restores cognitive motivation in Parkinson's disease. *Brain* **142**, 719–732 (2019).
10. Torta, D. M. E. *et al.* On the role of dopamine replacement therapy in decision-making, working memory, and reward in Parkinson's disease: does the therapy-dose matter?. *Brain Cogn.* **71**, 84–91 (2009).
11. Sharp, M. E. *et al.* Decisions under risk in Parkinson's disease: preserved evaluation of probability and magnitude. *Neuropsychologia* **51**, 2679–2689 (2013).
12. Voon, V. *et al.* Dopamine agonists and risk: Impulse control disorders in Parkinson's; Disease. *Brain* (2011). doi:10.1093/brain/awr080
13. M., B. *et al.* Gambling behavior in Parkinson's Disease: Impulsivity, reward mechanism and cortical brain oscillations. *Psychiatry Res.* **270**, 974–980 (2018).
14. Balasubramani, P. P. *et al.* Identifying the basal ganglia network model markers for medication-induced impulsivity in Parkinson's disease patients. *PLoS One* **10**, e0127542 (2015).
15. Bentivoglio, A. R., Baldonero, E., Ricciardi, L., De Nigris, F. & Daniele, A. Neuropsychological features of patients with Parkinson's disease and impulse control disorders. *Neurol. Sci.* (2013). doi:10.1007/s10072-012-1224-5
16. J.W., B. *et al.* Iowa Gambling Task Performance in Parkinson Disease Patients with Impulse Control Disorders. *Arch. Clin. Neuropsychol.* **34**, 310–318 (2019).

17. Bodi, N. *et al.* Reward-learning and the novelty-seeking personality: a between- and within-subjects study of the effects of dopamine agonists on young Parkinson's patients. *Brain* **132**, 2385–2395 (2009).
18. Buelow, M. T. *et al.* The contribution of apathy and increased learning trials to risky decision-making in Parkinson's disease. *Arch. Clin. Neuropsychol.* **29**, 100–109 (2014).
19. A., C. *et al.* Iowa gambling task impairment in Parkinson's disease can be normalised by reduction of dopaminergic medication after subthalamic stimulation. *J. Neurol. Neurosurg. Psychiatry* **86**, 186–190 (2015).
20. Cavanagh, J. F. *et al.* Cognitive states influence dopamine-driven aberrant learning in Parkinson's disease. *Cortex* (2017). doi:10.1016/j.cortex.2017.02.021
21. V., C. *et al.* Motivation, reward, and Parkinson's disease: Influence of dopatherapy. *Neuropsychologia* **40**, 2257–2267 (2002).
22. Delazer, M. *et al.* Decision making under risk and under ambiguity in Parkinson's disease. *Neuropsychologia* (2009). doi:10.1016/j.neuropsychologia.2009.02.034
23. Euteneuer, F. *et al.* Dissociation of decision-making under ambiguity and decision-making under risk in patients with Parkinson's disease: A neuropsychological and psychophysiological study. *Neuropsychologia* (2009). doi:10.1016/j.neuropsychologia.2009.06.014
24. Evens, R. *et al.* The impact of Parkinson's disease and subthalamic deep brain stimulation on reward processing. *Neuropsychologia* (2015). doi:10.1016/j.neuropsychologia.2015.05.005
25. S., G. *et al.* Cortical and striatal reward processing in Parkinson's disease psychosis. *Front. Neurol.* **8**, 156 (2017).
26. Gescheidt, T. *et al.* Iowa Gambling Task in patients with early-onset Parkinson's disease: strategy analysis. *Neurol. Sci.* **33**, 1329–1335 (2012).
27. Graef, S. *et al.* Differential influence of levodopa on reward-based learning in Parkinson's disease. *Front. Hum. Neurosci.* **4**, 169 (2010).
28. Herzallah, M. M. *et al.* Depression Reduces Accuracy While Parkinsonism Slows Response Time for Processing Positive Feedback in Patients with Parkinson's Disease with Comorbid Major Depressive Disorder Tested on a Probabilistic Category-Learning Task. *Front. psychiatry* **8**, 84 (2017).
29. Housden, C. R. *et al.* Intact reward learning but elevated delay discounting in Parkinson's disease patients with impulsive-compulsive spectrum behaviors. *Neuropsychopharmacology* **35**, 2155–2164 (2010).
30. Ibarretxe-Bilbao, N. *et al.* Neuroanatomical correlates of impaired decision-making and facial emotion recognition in early Parkinson's disease. *Eur. J. Neurosci.* (2009). doi:10.1111/j.1460-9568.2009.06892.x
31. Kobayakawa, M., Koyama, S., Mimura, M. & Kawamura, M. Decision making in Parkinson's disease: Analysis of behavioral and physiological patterns in the Iowa Gambling task. *Mov. Disord.* (2008). doi:10.1002/mds.21865
32. Kobayakawa, M., Tsuruya, N. & Kawamura, M. Sensitivity to reward and punishment in Parkinson's disease: An analysis of behavioral patterns using a modified version of the Iowa gambling task. *Park. Relat. Disord.* (2010). doi:10.1016/j.parkreldis.2010.04.011
33. Mapelli, D., Di Rosa, E., Cavalletti, M., Schiff, S. & Tamburin, S. Decision and dopaminergic system: an ERPs study of Iowa gambling task in Parkinson's disease. *Front. Psychol.* **5**, 684 (2014).
34. Martínez-Horta, S., Pagonabarraga, J., Fernández De Bobadilla, R., García-Sanchez, C. & Kulisevsky, J. Apathy in Parkinson's disease: More than just executive dysfunction. *J. Int. Neuropsychol. Soc.* (2013). doi:10.1017/S1355617713000131
35. Mimura, M. *et al.* Impaired decision-making in Parkinson's disease. *Parkinsonism Relat. Disord.* **12**, 169–175 (2006).
36. Pagonabarraga, J. *et al.* Controlled study of decision-making and cognitive impairment in Parkinson's disease. *Mov. Disord.* **22**, 1430–1435 (2007).
37. Paz-Alonso, P. M. *et al.* Functional inhibitory control dynamics in impulse control disorders in Parkinson's disease. *Mov. Disord.* **35**, 316–325 (2020).

38. Perretta, J. G., Pari, G. & Beninger, R. J. Effects of Parkinson Disease on Two Putative Nondeclarative Learning Tasks. *Cogn. Behav. Neurol.* (2005). doi:10.1097/01.wnn.0000187939.81541.1d
39. Pignatti, R. *et al.* Selective IGT decision-making impairment in a patient with juvenile Parkinson's disease and pathological gambling: A role for dopaminergic therapy? *Neurocase* (2012). doi:10.1080/13554794.2011.633529
40. Pineau, F. *et al.* Executive functioning and risk-taking behavior in Parkinson's disease patients with impulse control disorders. *J. Neural Transm.* **123**, 573–581 (2016).
41. D., H. *et al.* Impulse control disorders in Parkinson's disease are associated with dysfunction in stimulus valuation but not action valuation. *J. Neurosci.* **34**, 7814–7824 (2014).
42. Poletti, M. *et al.* Decision making in de novo Parkinson's disease. *Mov. Disord.* (2010). doi:10.1002/mds.23098
43. Poletti, M. *et al.* Alexithymia may modulate decision making in patients with de novo Parkinson's disease. *Funct. Neurol.* **26**, 127–131 (2011).
44. Rossi, M. *et al.* Decision-making in Parkinson's disease patients with and without pathological gambling. *Eur. J. Neurol.* **17**, 97–102 (2010).
45. Saez-Francas, N. *et al.* Relationship between poor decision-making process and fatigue perception in Parkinson's disease patients. *J. Neurol. Sci.* **337**, 167–172 (2014).
46. Thiel, A. *et al.* Activation of basal ganglia loops in idiopathic Parkinson's disease: A PET study. *J. Neural Transm.* (2003). doi:10.1007/s00702-003-0041-7
47. Timmer, M. H. M., Sescousse, G., Van Der Schaaf, M. E., Esselink, R. A. J. & Cools, R. Reward learning deficits in Parkinson's disease depend on depression. *Psychol. Med.* (2017). doi:10.1017/S0033291717000769
48. Xi, C. *et al.* Theory of mind and decision-making processes are impaired in Parkinson's disease. *Behav. Brain Res.* (2015). doi:10.1016/j.bbr.2014.11.035
49. E., Y. & S., A. Decision-making and impulse-control disorders in parkinson's disease: Influence of dopaminergic treatment. *Neurol. Sci. Neurophysiol.* **37**, 11–17 (2020).
50. Drew, D. S. *et al.* Dopamine and reward hypersensitivity in Parkinson's disease with impulse control disorder. *Brain* **143**, 2502–2518 (2020).
51. A.H., E. *et al.* Compulsive use of dopaminergic drug therapy in Parkinson's disease: reward and anti-reward. *Mov. Disord.* **25**, 867–876 (2010).
52. Lawrence, A. D., Goerendt, I. K., Brooks, D. J., A.D., L. & I.K., G. Apathy blunts neural response to money in Parkinson's disease. *Soc. Neurosci.* **6**, 653–662 (2011).
53. Muhammed, K. *et al.* Reward sensitivity deficits modulated by dopamine are associated with apathy in Parkinson's disease. *Brain* **139**, 2706–2721 (2016).
54. J.B., R. *et al.* Motivational engagement in Parkinson's disease: Preparation for motivated action. *Int. J. Psychophysiol.* **99**, 24–32 (2016).
55. M.H.M., T. *et al.* Enhanced motivation of cognitive control in Parkinson's disease. *Eur. J. Neurosci.* **48**, 2374–2384 (2018).
